# Proteomic Analysis of Estrogen-Mediated Signal Transduction in Osteoclasts Formation

**DOI:** 10.1155/2015/596789

**Published:** 2015-05-18

**Authors:** Qi Xiong, Peifu Tang, Yanpan Gao, Lihai Zhang, Wei Ge

**Affiliations:** ^1^Department of Orthopedics, General Hospital of Chinese PLA, Beijing 100853, China; ^2^National Key Laboratory of Medical Molecular Biology & Department of Immunology, Institute of Basic Medical Sciences, Chinese Academy of Medical Sciences, No. 5 Dongdansantiao, Dongcheng District, Beijing 100005, China

## Abstract

Estrogen plays an important role in inhibiting osteoclast differentiation and protecting against bone loss from osteoporosis, especially in postmenopausal women. However, the precise mechanisms underlying the effect of estrogen on osteoclasts are not well known. In the present study, we performed proteomics analysis and bioinformatics analysis to comprehensively compare the differential expression of proteins in receptor activator of nuclear factor-*κ*B ligand RANKL-induced osteoclasts in the presence and absence of estrogen. We identified 6403 proteins, of which 124 were upregulated and 231 were downregulated by estrogen. Bioinformatics analysis showed that estrogen treatment interfered with 77 intracellular pathways, including both confirmed canonical and unconfirmed pathways of osteoclast formation. Our findings validate the inhibitory effect of estrogen on osteoclasts via the promotion of apoptosis and suppression of differentiation and polarization and suggest that estrogen might inhibit osteoclast formation via other pathways, which requires further investigation and verification.

## 1. Introduction

Bone is a dynamic organ constantly renewed through a delicate balance between bone formation and resorption [1]. Abnormal bone resorption by osteoclasts leads to pathologically reduced bone mineral density, which is characteristic of osteolytic diseases, such as postmenopausal osteoporosis [2]. Estrogen is a key regulator of bone mass in postmenopausal osteoporosis. Estrogen deficiency in postmenopausal women frequently leads to osteoporosis, and estrogen replacement therapy is an effective means of retarding the decrease in bone density [3, 4]. Estrogen prohibits osteoclast bone resorption directly and indirectly. In direct regulation, estrogen receptors (ERs) *α* and *β* mediate most effects of estrogen. Estrogen activates ER*α*, but not ER*β*, reducing the already short lifespan of differentiated osteoclasts through apoptosis by activating the Fas cell surface death receptor (Fas)/Fas ligand (FasL) system, and induces apoptosis in a dose- and time-dependent manner [5–7]. In addition, estrogen suppresses osteoclast formation by downregulating Jun N-terminal kinase 1 (JNK1) and cellular-Jun (c-Jun) [8]. Moreover, estrogen decreases the expression of *β*3 integrin (ITGB3) and subsequently inhibits osteoclast adhesion [9]. However, the precise mechanism by which estrogen regulates osteoclasts remains unclear.

Osteoclasts are the only bone-resorbing cells derived from hematopoietic stem cells; they are induced by the receptor activator of nuclear factor-*κ*B (RANK) ligand (RANKL) and undergo differentiation and fusion, resulting in large multinucleated cells [10]. RANKL and RANK interaction induces intracellular cascades that are critical for osteoclast differentiation and activation [11]. RANKL promotes osteoclast differentiation via activation of the nuclear factor-*κ*B (NF-*κ*B) pathway [12]. Downstream signaling of RANK is mediated by the recruitment of tumor necrosis factor (TNF) receptor-associated factors (TRAFs). Of the TRAF family members, TRAF6 is crucial for RANKL-induced osteoclast formation. TRAF6 activates signaling components such as NF-*κ*B and mitogen-activated protein kinase (MAPK) [10, 13]. Moreover, RANKL stimulates the phosphorylation of microphthalmia-associated transcription factor (MITF), another transcription factor associated with osteoclastogenesis. RANK signaling also induces the transcription factor activator protein-1 (AP-1) by activating its component c-Fos [14]. NF-*κ*B and c-Fos activation leads to the induction of nuclear factor of activated T-cells, cytoplasmic, calcineurin-dependent 1 (NFATc1). Transcription factors such as NFATc1 and MITF form a transcription factor complex that stimulates the expression of osteoclast-specific genes such as cathepsin K (*CTSK*) and* TRAP *[15, 16]. RANKL also reduces the level of Fas expression in mature osteoclasts, thereby reducing Fas-mediated apoptosis [17].

Activation of bone resorption also involves osteoclast polarization and adhesion to the bone matrix. Osteoclasts polarize to form a tight sealing zone in which protons digest bone minerals; cathepsin K digests the organic matrix. Formation of the sealing zone requires integrin-mediated recognition of the extracellular matrix. Several studies have demonstrated that *α*v*β*3 integrin plays a central role in osteoclast adhesion [18, 19]. Moreover, it has been suggested that ITGB3 participates in the formation of the actin ring and normal ruffled border of osteoclasts [20]. In addition to integrin, small guanosine triphosphatases (GTPases), including RhoA, Rac, and Arf6 (ADP-ribosylation factor 6), are also key regulators of the formation of the sealing zone [21].

Estrogen is involved in a variety of cytokines that regulate osteoclast differentiation. Therefore, we performed proteomics analysis and bioinformatics analysis of the protein changes that occur when osteoclast precursor RAW 264.7 cells differentiate into mature osteoclasts following induction by RANKL in the presence or absence of estrogen to comprehensively investigate estrogen regulation of differentiating osteoclasts.

## 2. Materials and Methods

### 2.1. Reagents

Soluble mouse RANKL (462-TEC-010) was purchased from R&D Systems. 17*β*-Estradiol (E8875) and acid phosphatase leukocyte kits (387-1) were purchased from Sigma-Aldrich. Urea (17-1319-01) was obtained from GE Healthcare. EDTA-free protease inhibitor cocktail was obtained from Roche. To obtain lysis buffer, 8 M urea was mixed with the protease inhibitor cocktail. The TMT 6-plex Isobaric Label Reagent Set (90061) was purchased from Thermo Scientific. Trypsin/Lys-C Mix (V5072) was purchased from Promega. The bicinchoninic acid (BCA) Protein Assay Kit was obtained from Thermo Scientific (23227). Dithiothreitol (DTT) (17131801) and indole acetic acid (IAA) (RPN6302) were obtained from GE Healthcare.

### 2.2. Cell Culture

RAW 264.7 cell line was obtained from the Chinese Academy of Medical Sciences (Beijing, China). Osteoclast formation was performed as previously described by Vincent et al., with minor modifications [22]. Briefly, to generate mature multinucleated osteoclasts, RAW 264.7 cells (1.5 × 10^5^ cells/cm^2^) were cultured in *α*-minimum essential medium with fetal bovine serum in 6-well plates and incubated at 37°C in 5% CO_2_. Cells were divided into two groups by adding 30 ng/mL RANKL with or without 10^−8 ^M 17*β*-estradiol to the culture system, and both groups were in triplicate. The culture medium was refreshed every other day. On day 5, cells were confirmed by tartrate-resistant acid phosphatase (TRAP) staining and harvested, and the triplicates of each group were mixed and prepared for tandem mass tag (TMT) labeling.

### 2.3. TRAP Staining

Mature osteoclasts were defined as TRAP-positive cells containing three or more nuclei. TRAP staining was performed using the acid phosphatase leukocyte kit according to the manufacturer's instructions.

### 2.4. Sample Preparation and TMT Labeling

For TMT labeling, sample preparation and labeling were performed as described by Xiong et al. [23]. Cells were washed with cold phosphate-buffered saline three times, followed by the addition of lysis buffer. Cell lysates were clarified by centrifugation. Then, the supernatant of each group was obtained. Protein concentrations were evaluated using the BCA Protein Assay Kit. About 100 *μ*g protein from each group was incubated in 10 mM DTT at 50°C for 1 h. Next, protein was incubated with 25 mM IAA away from light for 2 h at room temperature. Subsequently, proteins were digested with the Trypsin/Lys-C Mix at a protein/protease ratio of 25 : 1 overnight at 37°C. The TMT isobaric label reagent set was used according to the manufacturer's manual to label proteins. Proteins extracted from cells cultured with RANKL in the presence or absence of 17*β*-estradiol were labeled with 0.8 mg TMT-129 or TMT-128, respectively. Equal amounts of the labeled protein digests from each group were combined for mass spectrometry (MS) analysis.

### 2.5. High Performance Liquid Chromatography (HPLC)

Fractionation of the combined protein digests was performed as described previously by van Ulsen et al. [24]. Briefly, the combined TMT-labeled samples were dissolved in 100 *μ*L 0.1% formic acid (FA) and translocated to MS tubes for HPLC (UltiMate 3000 UHPLC, Thermo Scientific). An Xbridge BEH300 C18 column (4.6 × 250 mm^2^, 5 *μ*m, 300 Å, Waters) was used. Fractions were collected every 1.5 min for each of the 50 microtubes. The fractions were dried in a vacuum concentrator at 37°C and then dissolved in 20 *μ*L 0.1% FA for further liquid chromatography (LC)-MS/MS analysis.

### 2.6. LC-MS/MS

A Q Exactive mass spectrometer was used for LC-MS/MS. Protein digests were separated by a 120 min gradient elution at a flow rate of 0.3 *μ*L/min using an UltiMate 3000 RSLCnano System (Thermo Scientific), followed by analysis using a directly interfaced Q Exactive Hybrid Quadrupole-Orbitrap Mass Spectrometer (Thermo Scientific). The analytical column was a homemade fused silica capillary column (75 *μ*m, 150 mm, Upchurch) packed with C18 resin (300 Å, 5 *μ*m, Varian). Xcalibur 2.1.2 software was used with the Q Exactive mass spectrometer in data-dependent acquisition mode. A single full-scan mass spectrum in Orbitrap (400–1800* m/z*, 60,000 resolutions) was followed by 10 data-dependent MS/MS scans at 27% normalized collision energy (HCD).

### 2.7. Data Analysis

Data were analyzed with the Thermo Scientific Proteome Discoverer software suite 1.3 with SEQUEST search engine and the mouse FASTA database from UniProt (released on July 9, 2014). In SEQUEST, full trypsin specificity was selected, two missed cleavages were allowed, carbamidomethylation (C) and TMT 6-plex (K and peptide N terminals) were set as the static modification, oxidation (M) was set as the dynamic modification, precursor ion mass tolerances were set at 20 ppm for all MS acquired in the Orbitrap mass analyzer, and the fragment ion mass tolerance was set at 20 mmu for all MS/MS spectra acquired. The ratio of proteins labeled with TMT-129 and TMT-128 was adjusted using the glyceraldehyde-3-phosphate dehydrogenase (GAPDH) ratio as the internal control. The downregulation and upregulation thresholds were set at 0.8 and 1.25, respectively. UniProtKB/Swiss-Prot accessions were converted into Entrez Gene IDs for subsequent analysis using the UniProt online ID mapping server. Another online analysis tool kit, bioDBnet, was used to transform the mouse gene IDs into homolog human gene IDs. The online PANTHER classification system was used to classify the proteins. The online WEB-based GEne SeT AnaLysis Toolkit (WebGestalt) was used to run enrichment analysis. Cytoscape 3.1.1 software and plug-ins were used to analyze the protein-protein interaction matching the WikiPathways database; significance was set at 0.0001.

## 3. Results

### 3.1. Effects of 17*β*-Estradiol on Osteoclast Formation

**Figure 1:**
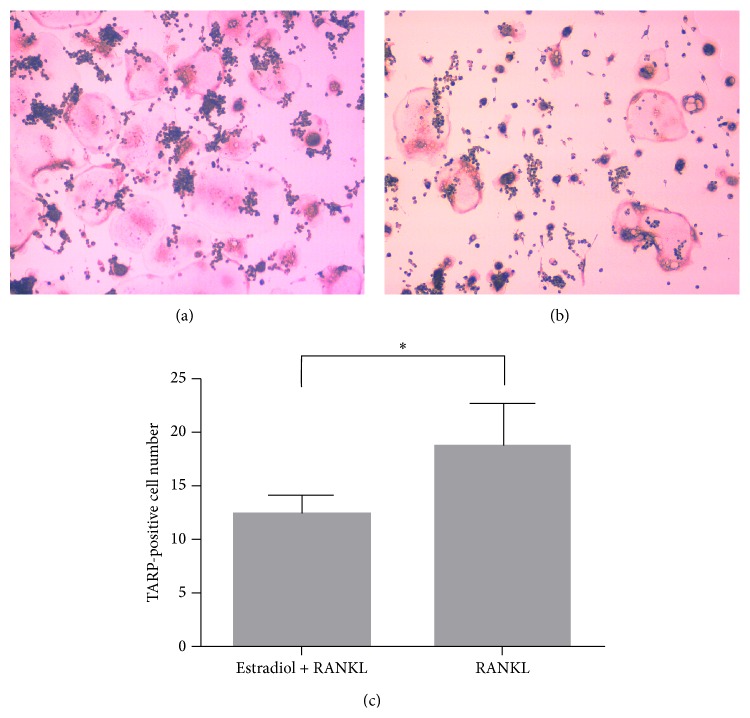
Estrogen inhibits osteoclast formation. (a) RAW 264.7 cells cultured with 30 ng/mL RANKL; (b) RAW 264.7 cells cultured with 30 ng/mL RANKL and 10^−8 ^M 17*β*-estradiol; (c) estrogen significantly reduced the number of TRAP-positive multinucleated osteoclasts. ^∗^ *P* < 0.05.

TRAP staining confirmed that RANKL efficiently induced the formation of mature osteoclasts on day 5. The number of TRAP-positive multinucleated osteoclasts in cells cultured with RANKL and 17*β*-estradiol was significantly less than that of cells cultured with RANKL only (Figure 1). Taken together, our data suggest that estrogen inhibits osteoclast formation* in vitro*.

### 3.2. Proteomics Analysis of Proteins in Estrogen-Treated Osteoclasts

To investigate the proteins contributing to the inhibition of multinucleated mature osteoclast formation, we conducted proteomics analysis to compare the differentially expressed proteins of RANKL-induced osteoclasts in the presence or absence of 17*β*-estradiol. We used Proteome Discoverer to assess the LC-MS/MS raw data. We identified a total of 6403 proteins, of which 124 were upregulated in osteoclasts cultured with RANKL and 17*β*-estradiol compared to cells cultured with RANKL only and 231 were downregulated when cells were cultured with RANKL and 17*β*-estradiol. Intriguingly, we could not obtain the ratio for four proteins (UniProt accession numbers: B1ARU1, B1ARU4, F7ACR9, and Q8CAQ8-2) (see upregulated and downregulated proteins in the Supplementary Material 1 available online at http://dx.doi.org/10.1155/2014/596789).

### 3.3. Identification and Classification of Identified Proteins

**Figure 2:**
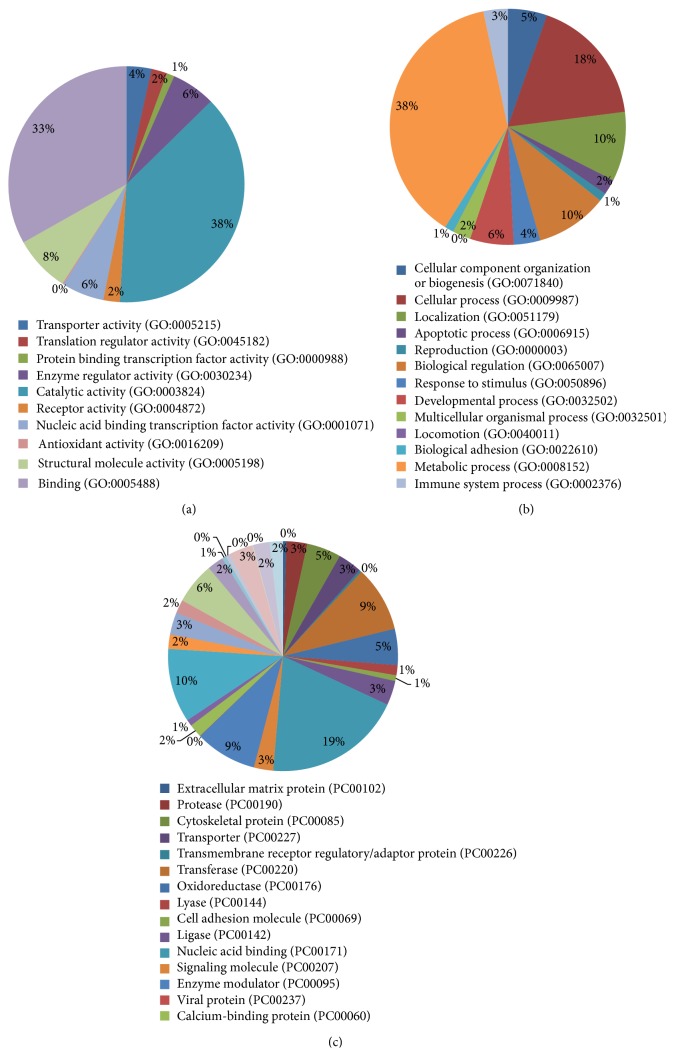
Classification of the identified proteins with PANNTHER. (a) Protein class hit against total PANTHER protein class. (b) Molecular function hit against total GO molecular function. (c) Biological process hit against total GO biological process.

To identify and classify the proteins, we first converted the UniProt accession profiles into recognizable gene IDs; we performed ID mapping using UniProt and the bioDBnet online analysis tool kit. Of the 6403 proteins identified, 5581 could be transformed into Entrez Gene IDs; 4853 mouse proteins homologous with those of humans were converted into human gene IDs. Thereafter, we classified the identified proteins using the PANTHER classification system. In total, 4450 proteins were matched to the total proteins in the PANTHER database, all of which were grouped into 29 subgroups, and the proteins identified were matched to 6442 biological processes in 13 categories. We also assessed the molecular functions of the identified proteins and identified 4464 molecular functions in 10 categories matched, where the proteins were mostly involved in catalytic activity and binding (Figure 2).

### 3.4. Bioinformatics Analysis of Identified Proteins

**Figure 3:**
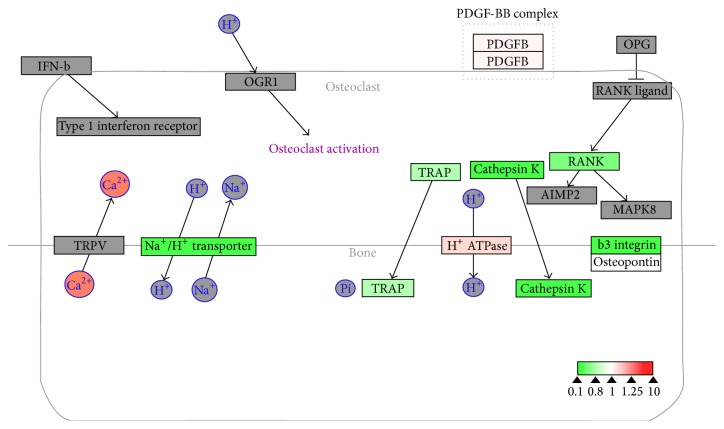
Estrogen regulates osteoclasts signaling pathway of osteoclasts. Grey boxes indicate proteins not identified in our study; green boxes indicate downregulated proteins; red boxes indicate upregulated proteins.

**Figure 4:**
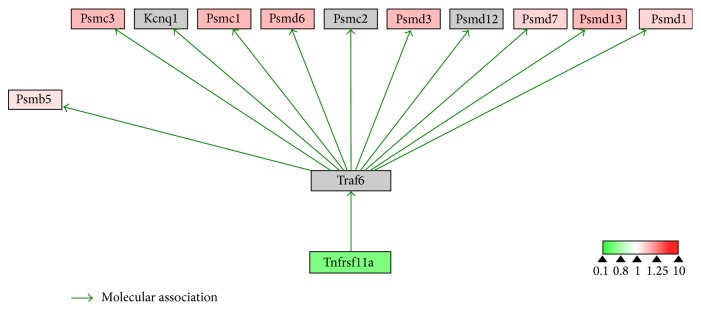
Estrogen regulates TNF-*α* NF-*κ*B signaling pathway of osteoclasts. Grey boxes indicate proteins not identified in our study; green boxes indicate downregulated proteins; red boxes indicate upregulated proteins.

**Figure 5:**
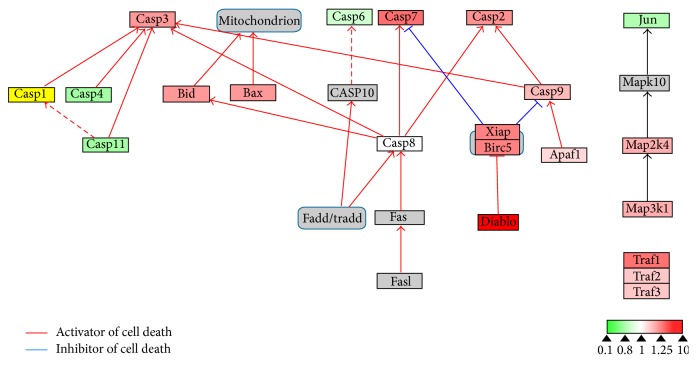
Estrogen regulates apoptosis signaling pathway of osteoclasts. Grey boxes indicate proteins not identified in our study; green boxes indicate downregulated proteins; red boxes indicate upregulated proteins.

**Figure 6:**
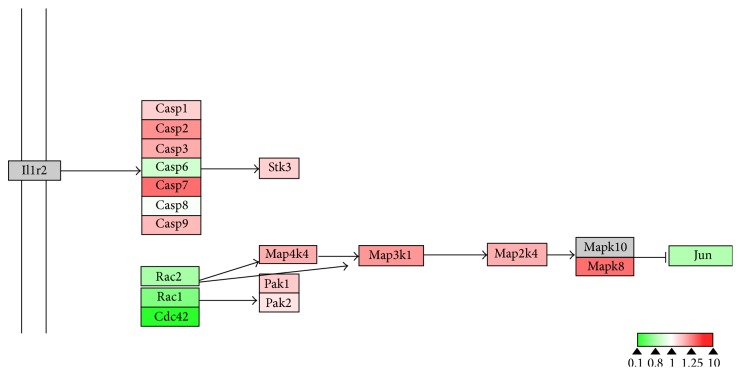
Estrogen regulates MAPK signaling pathway of osteoclasts. Grey boxes indicate proteins not identified in our study; green boxes indicate downregulated proteins; red boxes indicate upregulated proteins.

**Figure 7:**
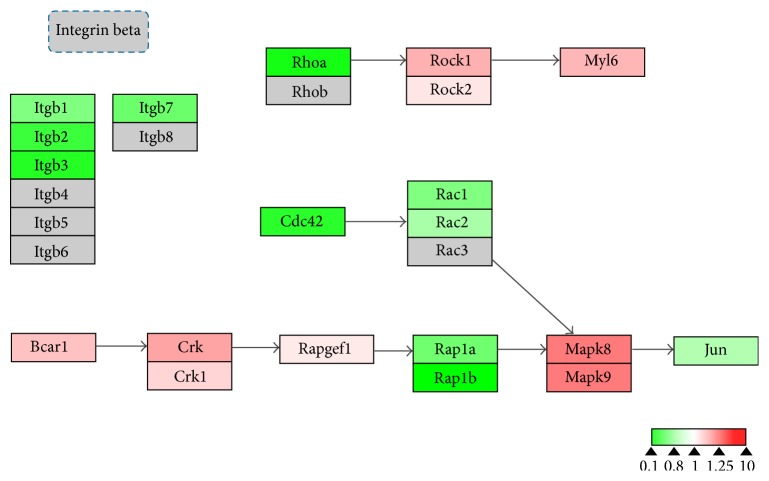
Estrogen regulates cell adhesion and polarization signaling pathway of osteoclasts. Grey boxes indicate proteins not identified in our study; green boxes indicate downregulated proteins; red boxes indicate upregulated proteins.

**Figure 8:**
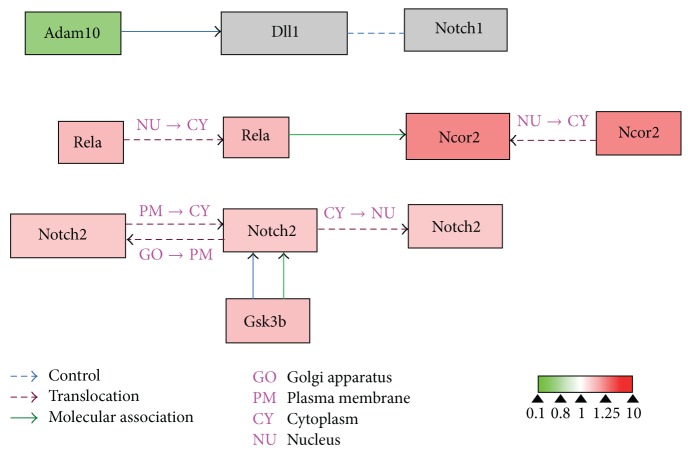
Estrogen regulates Delta-Notch signaling pathway of osteoclasts. Grey boxes indicate proteins not identified in our study; green boxes indicate downregulated proteins; red boxes indicate upregulated proteins.

To evaluate the effects of 17*β*-estradiol on RANKL-induced osteoclast differentiation, we performed bioinformatics analysis using WebGestalt and Cytoscape. WebGestalt was used to conduct enrichment analysis based on the WikiPathways database: 2770 of the 6403 proteins participating in 77 pathways were mapped with high confidence; of these, we found pathways confirmed as being involved in osteoclast differentiation, including pathways for TNF-*α*/NF-*κ*B signaling, focal adhesion, estrogen signaling, apoptosis, MAPK cascade, senescence, autophagy, FAS, stress induction of heat shock protein (HSP) regulation, and the osteoclast signaling. Moreover, we identified pathways that have not been verified as being associated with osteoclasts to date, including the pathways for Delta-Notch signaling, urea cycle, and metabolism of amino groups (Figures 3–8). Next, we used Cytoscape to analyze the differentially expressed proteins in osteoclasts cultured with 17*β*-estradiol and RANKL compared to osteoclasts cultured with only RANKL in the pathways identified using WebGestalt. The proteins were mapped to WikiPathways, and we observed that protein expression was altered in a variety of pathways. In the osteoclast signaling pathway, it was apparent that the hallmarks of osteoclast differentiation, such as TRAP, cathepsin K, and RANK, were downregulated in an obvious manner in cells cultured with RANKL and 17*β*-estradiol. These findings indicate that osteoclast differentiation was inhibited. However, we found that H^+^-ATPase was slightly upregulated, indicating more bone resorption (Figure 3). Furthermore, we observed in the TNF-*α*/NF-*κ*B signaling pathway that TNF receptor superfamily, member 11a, NF-*κ*B activator (TNFRSF11A or RANK), downstream of which was Traf6, was downregulated by 17*β*-estradiol (Figure 4). In addition, we screened the Fas and apoptosis pathways and found that caspase-3 (CASP3), which promotes apoptosis, was upregulated (Figure 5). CASP3 was also upregulated in the MAPK pathway, a known pathway essential for osteoclasts (Figure 6). Furthermore, we assessed the pathway associated with osteoclast polarization, finding that RhoA was obviously upregulated (Figure 7). Moreover, we were surprised to find that Jun was downregulated in almost all of the above-mentioned pathways. Taken together, our results suggest that 17*β*-estradiol promotes osteoclast apoptosis and inhibits osteoclast differentiation and polarization; however, our results demonstrate that 17*β*-estradiol induces osteoclast bone resorption slightly.

## 4. Discussion

In this study, we used proteomics analysis to comprehensively screen differentially expressed proteins and bioinformatics analysis to thoroughly evaluate the pathways and biological processes in RANKL-induced osteoclast formation in the presence or absence of 17*β*-estradiol. Consistent with previous studies [25, 26], our findings indicate that estrogen significantly inhibits TRAP-positive multinucleated osteoclasts. We identified 6403 proteins in 29 categories in the osteoclasts, of which 124 were upregulated and 231 were downregulated by estrogen stimulation. All identified proteins are involved in 77 signaling pathways in 13 biological processes. Estrogen probably prohibited the formation of mature osteoclasts by interfering with these intracellular signals. Our results provide comprehensive verification and identification of the effects of estrogen on osteoclasts.

The OPG/RANKL/RANK signaling pathway is a fundamental regulatory system for osteoclastogenesis. Therefore, it is reasonable to speculate whether estrogen inhibits osteoclast formation through this pathway. Palacios et al. demonstrated that estrogen directly binds with ER*α*, a membrane receptor expressed on osteoclast precursors, and decreases the formation of TRAP-expressing osteoclasts [27]. Cathepsin K plays critical roles in osteoclast bone resorption; Shi et al. reported that cathepsin K was increased in an animal model of postmenopausal osteoporosis [28]. In our study, we determined that RANK, TRAP, and cathepsin K were downregulated in an obvious manner in the osteoclast pathway via WikiPathways database matching. Our findings confirmed estrogen inhibition of osteoclast formation at the molecular level. However, our results showed that H^+^-ATPase expression was increased when osteoclasts were treated with estrogen. H^+^-ATPase is required in proton secretion by the ruffled border of activated osteoclasts to maintain a low-pH microenvironment of the resorption lacunae and mediates bone resorption [29]. Thus, estrogen might somehow enhance osteoclast bone resorption, though the mechanisms have not been elucidated. Taken together, these results strongly support the premise that estrogen reduces the formation of mature osteoclasts by regulating the osteoclast signaling pathways.

As the downstream signaling molecules of RANK include NF-*κ*B and TRAF6 [30–32], we compared their differential expression in the NF-*κ*B pathway between osteoclasts cultured with and without estrogen. In the TNF-*α*/NF-*κ*B signaling pathway mapped in the WikiPathways database, estrogen decreased the expression of membrane receptor TNFRSF11A. As expected, we did not detect TRAF6, a downstream molecule of TNFRSF11A. Considering the fact TRAF6 initiates the NF-*κ*B signaling cascade, and TRAF6 deficiency results in reduced osteoclast differentiation or function, we believe that estrogen inhibition of osteoclast formation is partially attributed to downregulation of the NF-*κ*B pathway.

Previous studies have reported that estrogen promotes osteoclast apoptosis. Saintier et al. demonstrated that estrogen induced nuclear condensation and increased Bax, CASP3, and CASP9 protein expression without altering BCL2 protein expression and* FASL* and* FAS* mRNA expression in osteoclasts, eventually inducing osteoclast apoptosis [33]. However, Nakamura et al. showed that estrogen induced osteoclast apoptosis by upregulating FasL expression [5]. Consistent with Saintier et al., we did not observe changes in BCL2 expression in osteoclasts cultured with estrogen, whereas Jun was downregulated in the upstream region of the antiapoptotic gene. Moreover, similar to Nakamura et al., we found that estrogen upregulated the Fas pathway; molecules downstream the pathway, that is, FAF1 (Fas-associated factor 1) and CASP3, both of which initiate apoptosis, were increased. To confirm the induction of apoptosis by estrogen, we matched our results with the apoptosis pathway in the WikiPathways database. As expected, proapoptotic proteins such as Bax, CASP3, and CASP9 were upregulated. In view of these findings, estrogen inhibits osteoclast formation by inducing apoptosis and reducing osteoclast lifespan.

Osteoclast bone resorption requires polarization, which is reorganization of the cytoskeleton and the formation of the actin-rich sealing zone [21]. In osteoclasts, these processes depend on vitronectin, ITGB3, and small GTPases, including RhoA, Rac, and Cdc43 [21, 34]. Zhang et al. reported that RhoA inhibition disrupted sealing zone formation and inhibited osteoclast formation [35]. Furthermore, other researchers have suggested that RhoA affects only the sealing zone, but not actin belt formation [36]. To the best of our knowledge, we are the first to suggest that estrogen affects RhoA and Rac expression in osteoclast formation. In the focal adhesion pathway, we found that RhoA, Rac1, and Rac2 were downregulated. Previous studies have indicated that RhoA alone was not sufficient to induce sealing zone formation [37, 38]. ITGB3 also plays an important role in regulating osteoclast function. Inhibition of ITGB3* in vitro* decreased the ability of osteoclasts to bind and degrade bone and promoted osteoclast apoptosis [18, 39, 40]. There is evidence that estrogen reduces ITGB3 expression in differentiating and mature osteoclasts in humans and mice and inhibits osteoclast adhesion [9, 33]. To confirm the effect of estrogen on integrin-mediated osteoclast adhesion, we further analyzed the differential expression of proteins in the integrin-mediated cell adhesion pathway. We found that ITGB1–3, ITGB7, ITGA5, and ITGAX were downregulated by estrogen. Therefore, our findings suggest that osteoclast adhesion capacity is decreased in the presence of estrogen through GTPase and integrin.

In addition to the verified pathways related to osteoclast formation, we determined that estrogen might interfere with other osteoclast signaling pathways, including the pathways for Delta-Notch signaling, urea cycle, and amino group metabolism, where Delta-Notch signaling plays a crucial role in cell-cell communication and cell fate decisions and coordinates with vascular endothelial growth factor (VEGF) pathways in upstream activating stimulus for angiogenesis [41]. Early studies have suggested that osteoclasts stimulate angiogenesis* in vitro* and* in vivo *[42, 43]. To our knowledge, however, whether Delta-Notch signaling participates in osteoclast formation has never been reported. In the present study, we observed that a variety of proteins were differentially expressed in the Delta-Notch signaling pathway: estrogen increased Notch2 and RelA while reducing ADAM metallopeptidase domain 10 (ADAM10). Although the pathways identified in this study require confirmation, our findings shed new light on the mechanisms of estrogen regulation of osteoclasts.

In conclusion, estrogen inhibits osteoclast formation by regulating the cell differentiation, apoptosis, adhesion, and other pathways. Additionally, estrogen might be involved in interference with other intracellular or intercellular pathways, although their precise mechanisms require future validation. Our results also shed new light on the development of more efficient therapeutic approaches for treating postmenopausal osteoporosis.

## Supplementary Material

Proteomic analysis of estrogen mediated upregulatied and downregulated proteins. Details of proteins were supplied, including accession numbers, description of the proteins, original ratio values and GAPDH adjusted values, scores and coverage.
